# Mir 145/143: tumor suppressor, oncogenic microenvironmental factor or …both?

**DOI:** 10.18632/aging.100965

**Published:** 2016-05-18

**Authors:** Mario Cioce, Sabrina Strano, Paola Muti, Giovanni Blandino

**Affiliations:** Oncogenomic and Epigenetic Unit, Regina Elena, National Cancer Institute, Rome, 00144 Italy

**Keywords:** miR-143/145, cancer, tumor suppressor, microenvironment

An interesting debate is emerging from the recent literature regarding the role of mir-143/145 in tumor initiation/progression. Herein, we aim to contribute our experience and observations to this ongoing debate. We will start from the experimental data presented in the outstanding work of the Tyler Jacks lab, by Dimitrova, Gocheva and colleagues [6]. They show, in a kRAS/p53 murine model of lung adenocarcinoma, that the contribution of the microRNA 143/145 cluster to lung cancer development is non-cell autonomous with the expression of mir143/145 from the tumor micro-environment resulting protumorigenic. More specifically, higher expression of microRNA 143/145 by endothelial cells would be pivotal in tumor progression by favoring tumor angiogenesis in a lung tissue specific fashion. The authors conclude, and their conclusion is shared by Almeida and Calin in a commentary on Genome Biology [1], that mir-143/145 is a non-cell autonomous oncogenic factor rather than a tumor suppressor, with their speculation further supported by the absence of tumor development in mice devoid of such microRNAs and by their lack of expression in murine epithelial cell lines [6]. This is in apparent contrast to what was published by our group and by many others, who provided evidence for the roles that mir-143/145 play as tumor suppressors in human tumors of epithelial origin, including and not limited to cervical, colon, gastric, breast and pancreatic carcinomas, NSCLC and malignant pleural mesothe-lioma (reviewed in Das and Pillai, 2015) [5].

In their commentary, Almeida and Calin claim that the “heterogeneity” of the human tumors collected for the human studies has prevented to provide a precise definition of the mir143/145 role. They basically substantiate such observation with the possibility that, in unfractionated human tumor tissues, residual expression of mir-143/145 by stromal component may escape the analysis of unfractionated human tumors. Even though this is certainly possible, we will try to provide a “parallel” and not mutually exclusive vision to integrate the ongoing discussion, starting from the work by Dimitrova et al. [6].

First, the data supporting a non-cell autonomous oncogenic role for the mir143/145 derive from a murine system. Dimitrova and coworkers employed an excellent albeit limited experimental system. In fact, while mice were engineered to express/not express specific tumor suppressors or oncogenes, represent an invaluable tool to study tumor progression, however there is little doubt left that such a system may reflect, at its best, one or few subtypes of its human counterparts of which it may recapitulate a gross history. Second, the mentioned data stem from the use of transgenic mice (Kras^G12D/+,^ p53^−/−^). Such a model basically represents a “frozen status”, again, corresponding only to a specific subset of the modeled tumors and “addicted” to the absence or expression of specific molecular lesions. Thus, engineered mice may not adequately represent the interpatient heterogeneity of human lung tumors. From this perspective, heterogeneity, more than representing an “Achille's Heel” of the mir-143/145 studies in human tumors, may represent an added welcome level of complexity toward understanding the “real life“ modulation of such a miRNA locus. Third, by manipulating the levels of microRNA 143/145 into MEFs (Mouse Embryo Fibroblasts), Dimitrova and coworkers conclude that no tumor suppressor activity can be ascribed to the microRNA 143/145 in such cells. Now, it is pleonastic to note that MEFs represent a totally different experimental system from the human epithelial tumors, in terms of embryonal origin and histotype. Thus, it is far from appropriate to draw conclusions regarding functions of the microRNA143/145 locus in human epithelial tumors from experiments performed by inducing deletion of the microRNAs into murine cells of non–epithelial origin. Fourth, and here we come to addressing the system we and many others have employed recently, where matched human specimens have been used. In all the cases, deep downregulation of the miRNA-143-145 expression as compared to normal matched tissues was observed. This occurred in extremely different tumors in terms of history, tissue of origin, and aggressiveness. In fact, both miR-143 and miR-145 were broadly described as downregulated in a plethora of solid tumors, including and not limited to breast, lung, colon (n=43 matched tissues), prostate, the gastrointestinal system, ovary, cervix, head and neck, bladder, thyroid, pituitary and gonads, germ-cell tumors (GCTs), gallbladder cancer, renal cell carcinoma, osteosarcoma, and neuroblastoma, mesothelioma (reviewed in Das and Pillai, 2015) [5] and thymic epithelial tumors [9]. Notably in most of the work mentioned, matched normal vs tumor samples were analyzed and, despite the fact that the analyzed tumors were very different in terms of history, tissue of origin, aggressiveness, mir143/145 levels, were invariably lower in the transformed tissues.

Rather convincing evidence supporting the tumor suppressor role of mir-143/145 in human malignancies comes from data showing that the downregulation of mir-143/145 is dynamic and correlates with the history of the disease. For example, Slaby et al. showed that downregulation of mir-145 was deeper in relapsing ccRCC as compared to the primary tumor, and, even deeper, in their metastatic counterparts [16]. Along the same line, reduced expression of mir-145 strongly correlated with shorter disease-free survival in prostate and small cell carcinoma of the cervix [2, 10]. Interestingly, by using in situ hybridization of >100 formalin fixed matched breast cancer specimens, Sempere and coworkers found that mir-145 was strongly downregulated in breast tumor tissues with its expression higher in the vessels and myoepithelial compartment of normal breast tissues but strongly reduced in the same compartments within the transformed tissues. However, there are not in enough samples to draw statistical conclusions [14]. On an even broader perspective, a relatively large study on postmenopausal women has recently been published where lower levels of mir-145 in circulating leukocytes were clearly shown to represent prognostic indicators linked to breast cancer progression [12]. Furthermore, dynamic reduction of mir-143/145 staining was observed upon exposure of rats to cigarette smoke [11]. Now, if increased expression of the mir-145 in stromal/endothelial human components would be oncogenic, as shown by Dimitrova et al. in the murine experimental system, it appears slightly counterintuitive that its levels were progressively downregulated in relapsing disease, metastatic disease, or upon stress stimuli conferring clear protumorigenic properties. Indeed, all these processes require a dynamic rearrangement of stromal cell subpopulations and neo-angiogenesis and this may likely lead to detectable changes of microRNA 143/145 expression. Thus, there generally appears to be a profound difference between human and murine tissues.

On a different note, other evidence point to active control of mir-143/145 levels within epithelial tumors and cell lines, in a protumorigenic direction. For example, we have shown that the mir-143/145 promoter is actively hyper-methylated in mesothelioma cancer cell lines4 and that hyper-methylation of this promoter correlates with the progressive downregulation of the mir-143/145 in brain metastases of lung tumors, as opposed to primary lung tumors and, lastly, to normal lung tissue [7]. Furthermore, treatment with histone deacetylase inhibitors and de-methylating agents or ectopic restoration of the miRNA levels correlates with lack of tumorigenicity in vivo [7] and similar results were shown in Burkitt's lymphoma cells [8]. The tumor specimens used in the mentioned studies were composed for a large portion of tumor tissue (typically 85-90% of the total tissue) and represented the results of the analysis from many different institutions. Notably, in most of the mentioned studies, the levels of mir-143-145 were in no case undetectable but rather significantly lower than their matched normal tissues. In regards to the cell lines, it may be interesting to note that in vitro grown human cell lines are virtually devoid of any significant stroma component, due to the long adaptation to in vitro growth. Thus, the described dynamic downregulation of the mir-143-145 taking place in the cell lines of the mentioned work may represent an intrinsic property of the transformed epithelial cells.

On the other hand, Almeida and Calin suggest the use of FACS-sorted cell subpopulations and/or the use of laser microdissection to better study the function of microRNAs, in order to ensure cell subpopulations homogeneity. In support of the latter, it is worth mentioning that, in head and neck cancer, ALDH+ve/CD44^+ve^ cancer stem cells exhibited low levels of miR-145 [18]. Similarly, chemoresistant ABCG2 expressing glioma with decreased levels of the microRNAs 143/145 positively correlated with poor prognosis [15]. Thus, there is increasing evidence for a cell-subpopulation restricted expression of the microRNAs, possibly modulated by external stimuli affecting the progression of the disease. However, the current repertoire of cell surface markers generally used to purify cell subpopulations is still very limited in terms of specificity, and to be able to narrow down a pure, functionally homogeneous cell subpopulations with absolute specificity and enough sensitivity. Thus we note that, the purification of cell subpopulations based on the current repertoire of cell surface markers and/or functional assays may not favorably deal with the intrinsic tumor heterogeneity of the cell subpopulations composing the tumor, which takes place at a single cell level, as shown by single-cell RNAseq studies [3, 13].

Finally, the observation that increased miR-145 could force [17] differentiation of ES cells into the mesoderm and ectoderm lineages may reconcile with the observed expression of mir-145 into the lung endothelial cells of the vessels of the transgenic kRAS/p53 mice, given the mesodermal derivation of endothelial cells in mouse. We are of the opinion that the use of matched human tissues may constitute an experimental system capable of capturing the complex modulation of the miRNA 143/145 in human tumors and may complement the observations performed in a single mouse strain, engineered to express frequent mutations in a time- and space- restricted manner. Therefore, the precise definition of the mir-143/145 contribution to oncogenesis is still ongoing…
